# An Experimental Field Study of Delayed Density Dependence in Natural Populations of *Aedes albopictus*

**DOI:** 10.1371/journal.pone.0035959

**Published:** 2012-04-26

**Authors:** Rachael K. Walsh, Caitlin Bradley, Charles S. Apperson, Fred Gould

**Affiliations:** 1 Department of Entomology, North Carolina State University, Raleigh, North Carolina, United States of America; 2 University of North Carolina, Chapel Hill, North Carolina, United States of America; 3 Fogarty International Center, National Institutes of Health, Bethesda, Maryland, United States of America; University of Otago, New Zealand

## Abstract

*Aedes albopictus*, a species known to transmit dengue and chikungunya viruses, is primarily a container-inhabiting mosquito. The potential for pathogen transmission by *Ae. albopictus* has increased our need to understand its ecology and population dynamics. Two parameters that we know little about are the impact of direct density-dependence and delayed density-dependence in the larval stage. The present study uses a manipulative experimental design, under field conditions, to understand the impact of delayed density dependence in a natural population of *Ae. albopictus* in Raleigh, North Carolina. Twenty liter buckets, divided in half prior to experimentation, placed in the field accumulated rainwater and detritus, providing oviposition and larval production sites for natural populations of *Ae. albopictus*. Two treatments, a larvae present and larvae absent treatment, were produced in each bucket. After five weeks all larvae were removed from both treatments and the buckets were covered with fine mesh cloth. Equal numbers of first instars were added to both treatments in every bucket. Pupae were collected daily and adults were frozen as they emerged. We found a significant impact of delayed density-dependence on larval survival, development time and adult body size in containers with high larval densities. Our results indicate that delayed density-dependence will have negative impacts on the mosquito population when larval densities are high enough to deplete accessible nutrients faster than the rate of natural food accumulation.

## Introduction

In the field of population ecology, both density independent factors and density dependent factors can influence the dynamics and growth of a population [1]. Density-dependent factors can impact populations by both first order feedback (direct density-dependence) and second order feedback (delayed density-dependence) [2]–[4]. First order factors have direct impacts on the current population or generation of individuals, whereas second order factors have delayed impacts on the current generation or on future generations of the population.

Delayed density-dependence has been shown to cause population fluctuations in plant-herbivore systems [5]–[7] and is associated with predator-prey cycles [8], [9]. However, there has been a long standing debate over the power of various methods for detecting delayed density-dependence [10]–[13].

Most investigators have used time series analysis to determine the role of delayed density-dependence. For example, long-term data have provided evidence for delayed density-dependence in populations of the potato aphid, *Macrosiphum euphorbiae*, [14], southern pine beetle, *Dendroctonus frontalis*, [15], forest tent caterpillar, *Malacosoma disstria*, [16] and in the cohabitating common sardine, *Strangomera bentincki*, and populations of the anchovy, *Engraulis ringens* [17].

In the present study, we tested for delayed density-dependence, specifically the impact of a current generation on a future generation of natural populations of *Aedes albopictus*, the Asian tiger mosquito. Instead of using time series analysis, we conducted a manipulative field experiment. *Ae. albopictus* is a competent laboratory vector of at least 22 arboviruses [reviewed in 18], including dengue (DENV) and chikungunya viruses (CHIKV) [19], [20]. It is established throughout the United States where its larvae develop in both naturally occurring tree holes and artificial containers such as bird baths, tires, and buckets [21], [22]. Previous studies in the laboratory and semi-field conditions have shown that direct density-dependence can impact larval survival, larval development, and adult body size in container inhabiting mosquitoes [23]–[26].

Aspbury and Juliano studied effects of leaf litter previously exploited by *Ae. triseriatus* larvae on a subsequent cohort of larvae [27]. In the laboratory, they prepared small containers holding leaf litter to provide two different treatments, one treatment without larvae and one treatment with 25 neonate larvae. All larvae were allowed to reach pupation and both leaf litter treatments were kept in the laboratory for 70 days. After 70 days, all small containers were taken to the field and placed in either a treehole or tire; each contained multiple small caged replicates of both treatments. Twenty-five newly hatched larvae were then placed into each small cage and survival, development time, and adult size were compared between treatments. Aspbury and Juliano found that in treeholes, small cages with leaf litter previously exploited by *Ae. triseriatus* larvae yielded longer larval development time and smaller adult size in the following cohort of larvae compared to litter that had not been previously exploited by larvae. Although this study is an appropriate starting point to assess the effects of a prior cohort, this study system was an open system in the sense of allowing flow of water between treatments within each treehole, which may reduce the apparent impact of delayed density-dependence. More importantly, number of larvae and amount of food were determined arbitrarily by the investigators.

Delayed density-dependence in container inhabiting mosquitoes is expected when the natural density of larvae are removing accessible nutrients faster than they are replaced by liter accumulation, decomposer growth, and primary production by photosynthetic microorganisms. Notably, we have found no published studies of mosquito species that assesses the impact of naturally-occurring larval densities on delayed density-dependence even in semi-naturally occurring containers.

Because container-inhabiting mosquitoes typically feed on dead and living organic material, including microorganisms such as fungi, protozoans, and bacteria that grow on the container's surfaces, detritus on surfaces or within the water column [28], it is hard to replicate the naturally occurring environment in the laboratory. *Ae. albopictus* spend more time foraging when leaves are present in a container compared to containers with only water [29], [30]. These leaves provide extra surface for microorganism growth and possibly a superior food source. Other studies have shown that larvae which fed on animal detritus yields higher population growth, larval survival, and adult size when compared to those fed on plant detritus [31], [32]. Our goal was to examine delayed density dependence in environments that closely mimicked those that *Ae. albopictus* encounter naturally.

Our study took advantage of the fact that in urban environments, *Ae. albopictus* larvae often develop in artificial containers such as 5-gallon buckets [21]. We used buckets in the field that were experimentally divided in half to provide a method of testing for delayed density-dependence in natural populations. We compared the fates of neonate larvae placed in one side of the bucket that previously had naturally occurring larvae within it to the fates of larvae placed in the other half that had no previous larvae because naturally-laid eggs had been removed. The hypothesis of strong delayed density-dependence predicts that in comparison to the treatment in which eggs were removed, the treatment that had larvae in the previous cohort would yield longer larval development time, lower larval survival, and smaller adult body size for the following cohort of larvae.

## Materials and Methods

### Study Site

This study took place in Raleigh, Wake County, North Carolina (city population of 356,321), in a suburban area of the city (census.gov). Seven houses within the city were chosen as sites for the study. The mean distance between nearest neighbor houses was 5.6 kilometers and the farthest house was 21 kilometers from the center of the city. The study took place from June through September, 2009 when natural populations of *Ae. albopictus* are prevalent.

### General Experimental Design

Twenty liter paint buckets were used as the experimental containers. Six buckets were placed outside each house for a total of 42 buckets. Prior to initiating the experiment, the buckets were vertically divided in half with styrofoam insulation and all edges between the two sides were sealed with hot glue. This method allowed two treatments within each bucket and ensured that water, nutrients, and larvae could not pass between the two treatments. All buckets were carefully checked to ensure the partition was well sealed before placing the buckets at each experimental site. Buckets were checked throughout the investigation and only one was found to have water passing between the treatments; that bucket was omitted from the analysis.

Our investigation consisted of two sequential experiments. In the first six weeks we manipulated the buckets to produce the two treatments, and in the following weeks we tested the impact of the treatments on survival, development time, and adult body size of a newly introduced cohort of larvae.

### Treatment Production

During the first six weeks of the investigation the two treatments were produced within each bucket: a “larvae-present” treatment (**hereafter LP**) and “larvae-absent” treatment (**hereafter LA**). The buckets were placed at residences with 1 L of rainwater seeded in each side. All buckets had a hole drilled 1 inch from the top of the bucket on each treatment side to prevent rain water from overflowing the bucket. The buckets were left uncovered for five weeks to allow natural rainwater (no additional water was added by the authors) and detritus to accumulate in the buckets, and for microorganism growth on that detritus. The natural populations of *Ae. albopictus* laid eggs in the buckets during that time period.

The LA treatment was checked daily for eggs and larvae. Eggs on the sides of the buckets were killed and removed with a paper towel. The water was checked for any larvae, and the small numbers of larvae found during the 5-week period were removed.

In the LP treatment, eggs were allowed to hatch and larvae were left undisturbed through pupation. Pupae were collected daily and placed in individual plastic tubes with water. When adults emerged they were stored frozen in individual 1.5 mL microcentrifuge tubes. Subsequently, adults were identified to species and the right wing was measured as an indicator of adult body size. Wings were measured using QCapture Pro 6.0 software.

On the last day of week 5, all eggs were removed and killed from both sides of the bucket, and all larvae and pupae were removed. All larvae from the LP treatment were counted as they were removed to yield an estimate of the larval density for each bucket. The buckets were covered with a fine mesh cloth to prevent oviposition. All buckets were checked every other day for one week for any new larvae that hatched from missed eggs. Any larvae found were removed.

### Effects of treatments

To test for the impact of delayed density-dependence, the same numbers of first instar larvae were placed into both treatments within a given bucket and the bucket was recovered with the fine mesh cloth to prevent further oviposition. If there was delayed density dependence, we expected the new larvae placed in the half of the bucket that previously had larvae would perform more poorly. The buckets contained different numbers of larvae during the first 5 weeks of the experiment. We assumed that the natural density of larvae in the recent past is the best predictor of future natural larval density in a given bucket (see [33] on differential attractiveness of buckets for *Aedes* oviposition). Therefore, in order to better mimic the expected natural situation, the number of larvae placed in each bucket reflected its past history of infestation.

The number of first instars released in a bucket was determined by a three step calculation: 1) for each bucket, a ratio of the number of pupae on the last day of 5 weeks to the number of larvae counted the same day was calculated, 2) Ratios were averaged across all buckets across all sites to give us an overall estimate of the ratio of pupae to larvae for any given day, 3) the daily number of pupae averaged over the first 5 weeks for each bucket was divided by the average pupae to larvae ratio to provide a rough estimate of the average daily number of larvae for each bucket. This estimate of average daily number of larvae was used to set the number of first instars released into both treatments of a given bucket. First instar larvae were released in two different densities, the natural density, as calculated (referred to as 1×), and ten times the calculated density (referred to as 10×). The numbers of estimated larvae and actual larvae released in each bucket are shown in supplemental material (Table S1). At each house, half of the buckets received larvae at each density, and buckets were randomly assigned to a density. All further references to 1× and 10× densities refer to the post-treatment phase of the experiments.

The first instar larvae (New Orleans, LA strain) for the 2^nd^ part of the experiment were hatched in the laboratory. The colony was maintained at ∼28°C, ∼75% RH, and a photoperiod of 14 h light:10 h dark, including two twilight periods (60 min each). Larvae in both treatments were left undisturbed through pupation. Pupae were removed daily and allowed to develop to adults as described previously. We then compared larval development time, larval survival and adult wing length between the two treatments.

### Statistical Analysis

Statistics were computed using SAS Version 9.13 (SAS Institute, Inc., Cary, NC, U.S.A.). To test for treatment effects on development time we used a mixed model analysis of variance (ANOVA) with a main effect of *treatment*. To test the effects on wing length, we used a mixed model ANOVA with main effects of *density* (1× and 10×), *treatment* (LP and LA), and *density x treatment interaction*. For both analyses, *bucket* nested within *house* (for development time), *house x density* (for wing length), and *house* were considered to be random effects. Development time and wing length variables were measured as means from the cohorts of each bucket. To test the effects on larval survival, an additional effect of *log of estimated larval density* was added to the above model. Larval survival and female wing length had different variances for the two levels of density. Therefore, these response variables were modeled with the degrees of freedom divided by density group using the Kenward-Roger method [34], resulting in fractional degrees of freedom. In order to allow comparisons between treatments within each bucket, *treatment* was modeled as a repeated statement within *bucket*. For pairwise comparisons, we examined differences in least squares (LS) means of the dependent variables for the LP and LA treatments within each density. Two containers had a Student residual greater than 3.5 and had a large impact on the results; these were considered outliers and removed from the analysis [35].

Development time was measured as the number of days it took for the first instar larvae to pupate. Proportion survival was measured as the total number of larvae that pupated divided by the total number of larvae placed in that container. We found that many buckets had a proportion of larvae surviving higher than 1.0. This could have resulted from unsuccessful killing of all eggs at the end of the first 5 weeks or eggs being laid on the surface of the water [36]. The 1× containers had 20 buckets with survival higher than 1.0 and the 10× containers had 3 buckets with survival higher than 1.0. The extra larvae would be anticipated to have a larger impact on the 1× containers than 10× containers.

## Results

### Development time

**Figure 1:**
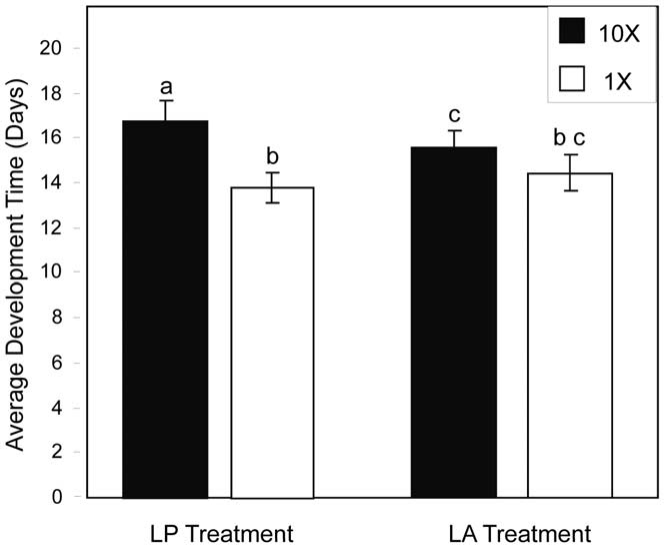
Results for development time. Least squares mean development time (days) for each treatment and density level. Vertical bars represent standard errors. Letters indicate significant differences.

There was not a significant effect of treatment on 1× density containers (*F*_1, 32_ = 0.51, *P* = 0.482), but there was a significant effect of treatment on 10× containers (*F*_1, 32_ = 9.58, *P* = 0.004) on development time. On average, larvae in 10× containers developed 8% more slowly in the LP treatment compared to the LA treatment (Figure 1).

There was no significant relationship between the log of estimated larval density and the difference in development time between the LP and LA treatments in a given container for either level of initial density (Figure S1).

### Survival

The mixed model analysis for proportion survival indicated a significant effect of log of estimated larval density (*F*_1, 19.8_ = 13.78, *P* = 0.001), density (*F*_1, 19.5_ = 10.62, *P* = 0.004), and a log of estimated larval density x treatment interaction (*F*_1, 19.2_ = 5.22, *P* = 0.033

**Figure 2:**
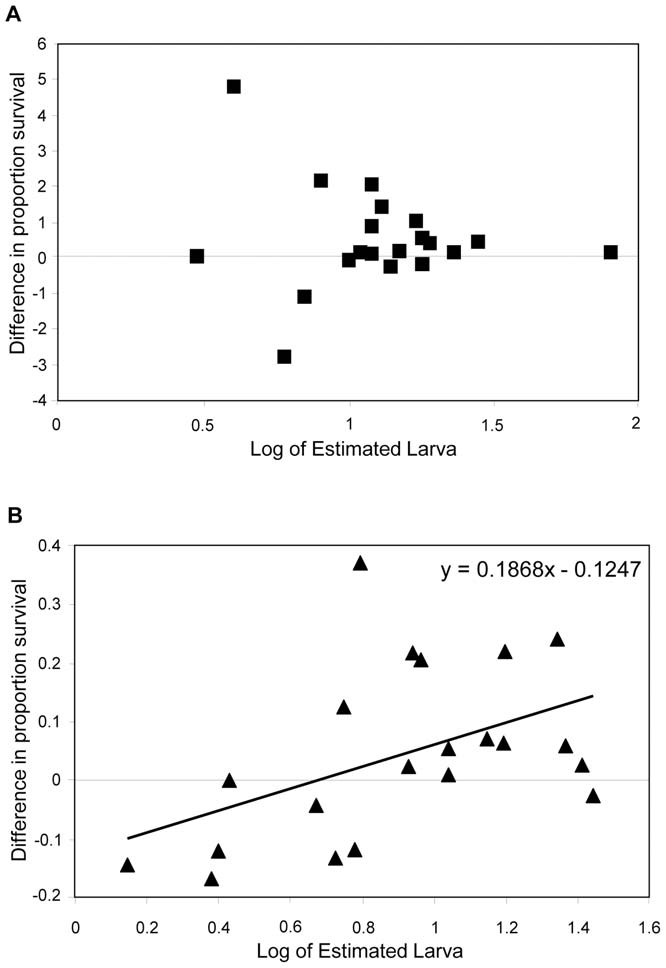
Effect of estimated larval density on proportion survival. Relationship between the difference in proportion survival between the LA treatment and LP treatment for each container and the log of estimated larval density for both 1× density containers (A) and 10× density containers (B).

Containers that had higher larval densities during the first 5 weeks of the experiment (pre-treatment) had a significantly lower proportion of larvae surviving to pupation and a larger difference in survival between the LP and LA treatments during the post-treatment phase. Using a linear regression, there was a significant relationship between the log of estimated larval density and the difference between the two treatments for proportion survival in 10× containers (*r^2^* = 0.223, df = 19, *P* = 0.031), but no significant relationship in 1× containers (*r^2^* = 0.019, df = 19, *P* = 0.567) (Figure 2).

### Male wing length

**Figure 3:**
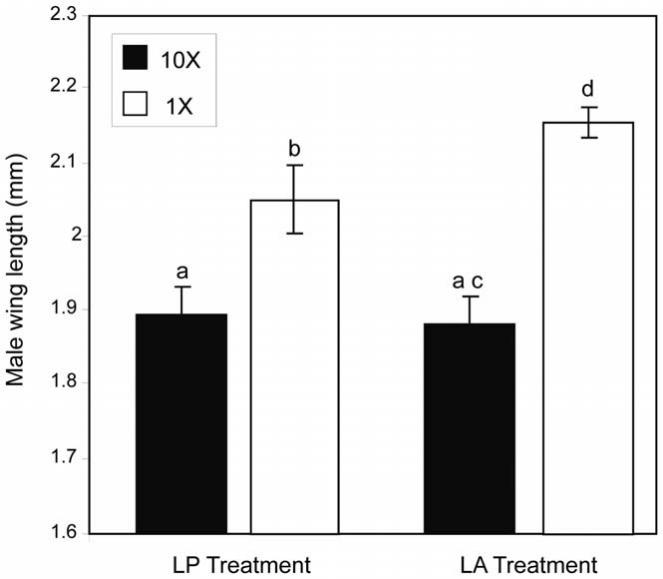
Results for male wing length. LS mean wing length (mm) for each treatment and density level. Vertical bars represent standard errors. Letters indicate significant differences.

Using a mixed model analysis, there was a significant effect of density (*F*_1, 6_ = 18.42, *P* = 0.005), treatment (*F*_1, 48_ = 5.93, *P* = 0.019), and a density by treatment interaction (*F*_1, 48_ = 8.23, *P* = 0.006) on male wing length. Males from containers with a 1× density had significantly longer wing lengths than males from 10× density containers across both treatments (*t*_6_ = 4.29, *P* = 0.005) (Figure 3).

**Figure 4:**
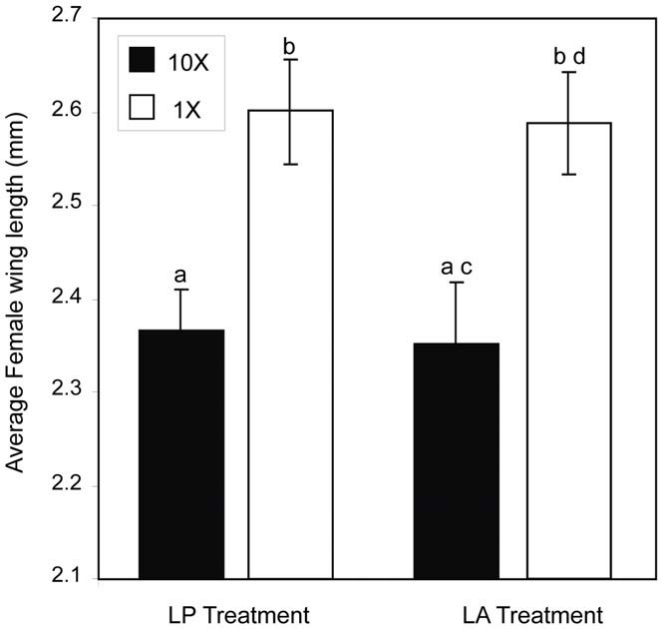
Results for female wing length. LS mean wing length (mm) for each treatment and density level. Vertical bars represent standard errors. Letters indicate significant differences.

There was a significant difference in male wing length between the LP treatment and the LA treatment for containers with 1× density (*t*_48_ = 3.54, *P* = 0.033). The average male wing length was larger for the LA treatment (mean = 2.16) than for the LP treatment (mean = 2.04) (Figure 4). There was no significant difference in male wing length between the two treatments for the 10× containers (*t*_48_ = 0.33, *P* = 0.75).

There was not a significant relationship detected between the log of estimated larval density (pre-treatment) and the difference in male wing length (post-treatment) between the two treatments for either the 10× density (*r^2^* = 0.046, df = 17, *P* = 0.393) or 1× density (*r^2^* = 0.149, df = 14, *P* = 0.155) (Figure S2).

### Female wing length

There was no significant effect of treatment (*F*_1, 29.3_ = 0.09, *P* = 0.767) or a density by treatment interaction on female wing length (*F*_1, 29.3_ = 0.01, *P* = 0.941) from the mixed model analysis. However, there was a significant effect of density on female wing length (*F*_1, 23_ = 11.61, *P* = 0.002) (Figure 4). On average, females that emerged from containers receiving 1× densities (mean = 2.56) had longer wing lengths than those that emerged from containers receiving 10× densities (mean = 2.36).

Similar to the results of male wing length, there was no significant relationship between the log of estimated larval density (pre-treatment) and the difference in female wing length (post-treatment) between the LP and LA treatments for either the 10× density (*r^2^* = 0.182, df = 15, *P* = 0.09) or 1× density(*r^2^* = 0.108, df = 14, *P* = 0.232) (Figure S3).

## Discussion

Containers with 10× densities had an overall lower proportion of larvae surviving to pupation and smaller wing lengths for both males and females. This suggests that direct density-dependence factors may influence larvae in field populations of *Ae. albopictus*. Although we cannot definitively conclude this from our data because we specifically added a cohort of neonates as opposed to testing for direct density-dependence on the naturally occurring larvae, our results are in agreement with previous laboratory [23] and semi-field condition studies [26], [37] of *Ae. albopictus*.

Delayed density-dependent effects were detected on larval survival to pupation when comparing the proportion of larvae surviving to pupation in the LP and LA treatments in containers receiving 10× densities. However, no significant effects of delayed density-dependence on survival were detected in containers receiving 1× densities.

Containers populated by greater numbers of naturally occurring larvae during the first five weeks (pre-treatment) yielded a stronger impact of delayed density-dependence on survival during the post-treatment phase as shown by the larger differences between the LP and LA treatments in containers that initially had high larval densities. These containers not only experienced higher numbers of naturally occurring larvae, but also had higher numbers of larvae released into the containers to test for delayed density-dependence because the numbers of larvae released were based on the naturally occurring larvae found in each container. Our results indicate that delayed density-dependent effects are produced at a larval density, which may be dependent on both previous and current cohorts, that depletes resources faster than the resources accumulate. It is likely that for this reason we did not detect delayed density-dependence in 1× containers. If populations cycle in the field, delayed density-dependence may only have impacts during the higher density points in the cycle when larvae cause a net decline in available nutrients for future cohorts.

Development time was negatively affected by delayed density-dependence, but only in 10× density containers. Consistent with our survival results, this finding further supports the hypothesis of delayed density-dependence acting only when a high larval density is reached. There was no impact of delayed density-dependence on female wing length. As seen with development time, delayed density-dependence negatively affected male wing length, but only in 1× containers. These results for male wing length are inconsistent with development time, in which delayed density-dependence was detected in 10× containers only. We analyzed male and female development time separately but there was no significant effect of treatment on development time for either sex to help explain the discrepancy in results.

One hypothesis for this discrepancy is there is a tradeoff between the rate of larval development and body size. Larvae that develop slower may have an opportunity to grow larger because as more time passes, the container is accumulating more food and because as time passes, more larvae may die, releasing survivors from competition. In the 1× containers, larvae developed faster and this could have impacted the difference between adult body sizes. In the LP treatment, the amount of food may have been sufficient for the larvae to pupate as fast as the LA treatment, but possibly not enough food for the adult sizes to be equivalent.

Gilpin and McClelland showed in laboratory studies that larvae starved for 40 days were able to pupate when liver powder was added to the system [38]. The ability to survive periods of time without food enables larvae to survive until the regeneration of food is able to occur within containers [39]. Both detritus and the microorganisms that feed on it, including bacteria, protozoa, and fungi, have been shown as an important food source for *Ae. albopictus* larvae and other container inhabiting mosquitoes [40]–[42]. Larval development could therefore also be dependent on the growth rate of microbial populations within the containers. However, we can not definitively test this or other hypothesis with data from our current experiment.

One limitation of this study is the inability to detect the amount of food and types of food present in the buckets. Different species of leaves decompose in water at different rates. Leaves that decompose at a more rapid rate support more larval mosquito growth compared to leaves that decompose slower [43]. It has also been shown that animal detritus decomposes faster than plant detritus and yields higher mosquito population growth [31], [32], [42]. The types and amounts of food likely varied between houses and the buckets within houses; therefore it probably impacted the presence and strength of delayed density-dependence detected in this study. While this is a limitation on our ability to explain the cause of delayed density dependence, this specific design enabled us to more closely estimate the degree of delayed density dependence that occurs in the field.

Our results are similar to those found by Aspbury and Juliano, the only other experiment assessing delayed density-dependence in a container–inhabiting mosquito in a field setting [27]. Both studies found an impact of delayed density-dependence on development time; however our study also detected an impact on larval survival. It is surprising that Aspbury and Juliano detected delayed density-dependence since they used an open flow water system between treatments. The small numbers of larvae used may have been appropriate for the size of the cages tested, but were not necessarily reflective of natural larval population densities. Our study was strengthened by the fact that the larval densities tested in our containers matched the naturally occurring densities assessed during the first part of the experiment. Using these natural larval densities, our study predicts that delayed density-dependence will only have a substantial impact on mosquito populations when the natural larval density is high enough that the available resources are depleted by the larvae faster than it is replenished by detritivores and microbes.

## Supporting Information

Figure S1 **Effect of estimated larval density on development time (days).** Relationship between the difference in development time between the LP treatment and LA treatment for each container and the log of estimated larval density for both 1× density containers (A) and 10× density containers (B).
(TIF)

Figure S2 **Effect of estimated larval density on male wing length (mm).** Relationship between the difference in male wing length between the LA treatment and LP treatment for each container and the log of estimated larval density for both 1× density containers (A) and 10× density containers (B).
(TIF)

Figure S3 **Effect of estimated larval density on female wing length (mm).** Relationship between the difference in female wing length between the LA treatment and LP treatment for each container and the log of estimated larval density for both 1× density containers (A) and 10× density containers (B).
(TIF)

Table S1 **Individual container information.** Table includes all information for each container including the estimated number of larvae during the pre-treatment phase, the number of larvae added for the post-treatment phase, and results for each parameter measured.
(XLS)
