# Supplementary figures and images for: An Experimental Field Study of Delayed Density Dependence in Natural Populations of Aedes albopictus
Source: PLoS One. 2012 Apr 26;7(4):e35959. doi: 10.1371/journal.pone.0035959 (PMC3338560; doi:10.1371/journal.pone.0035959)

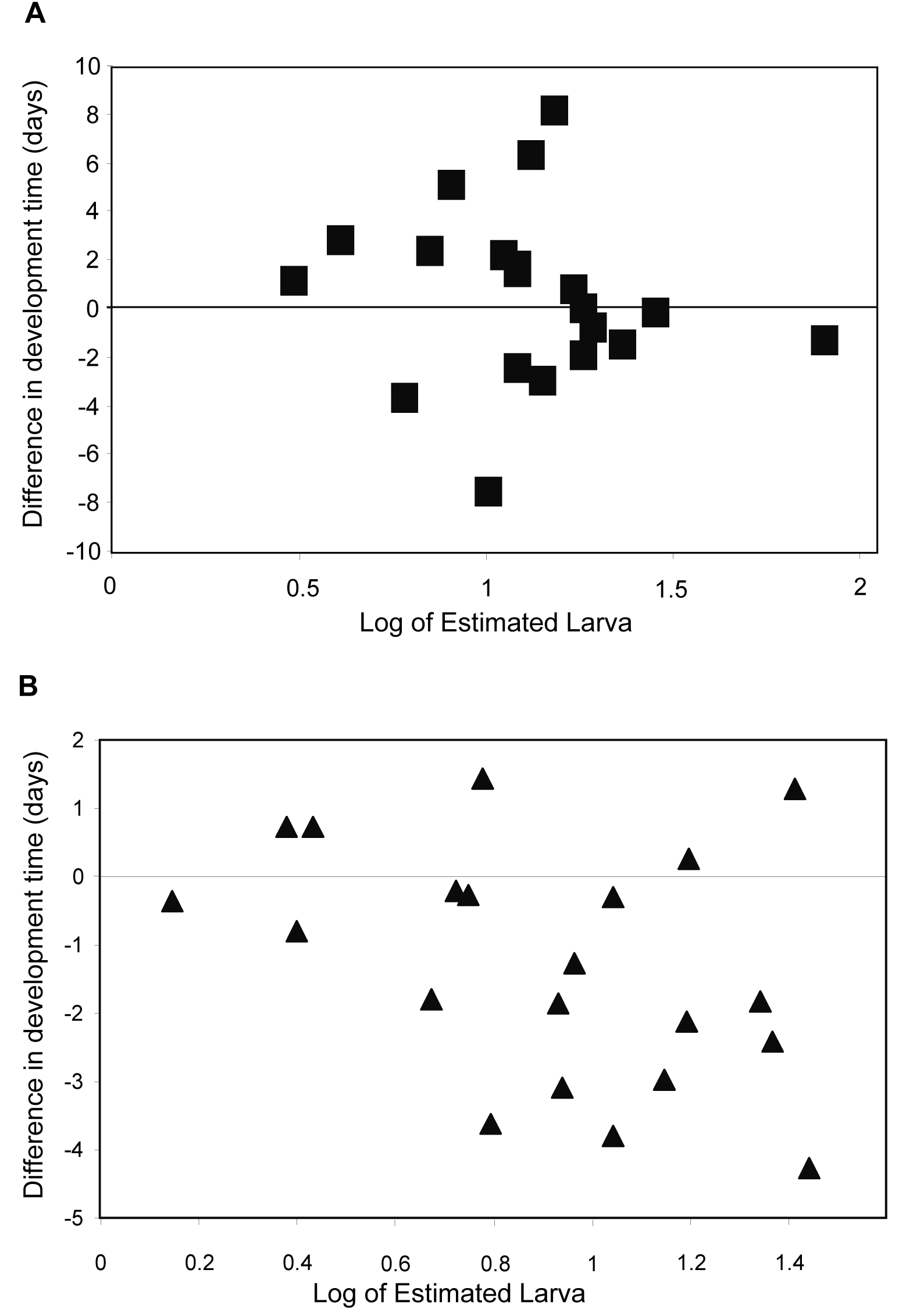

Supplement: Figure S1 — Effect of estimated larval density on development time (days). Relationship between the difference in development time between the LP treatment and LA treatment for each container and the log of estimated larval density for both 1× density containers (A) and 10× density containers (B). (TIF) [file pone.0035959.s001.tif]

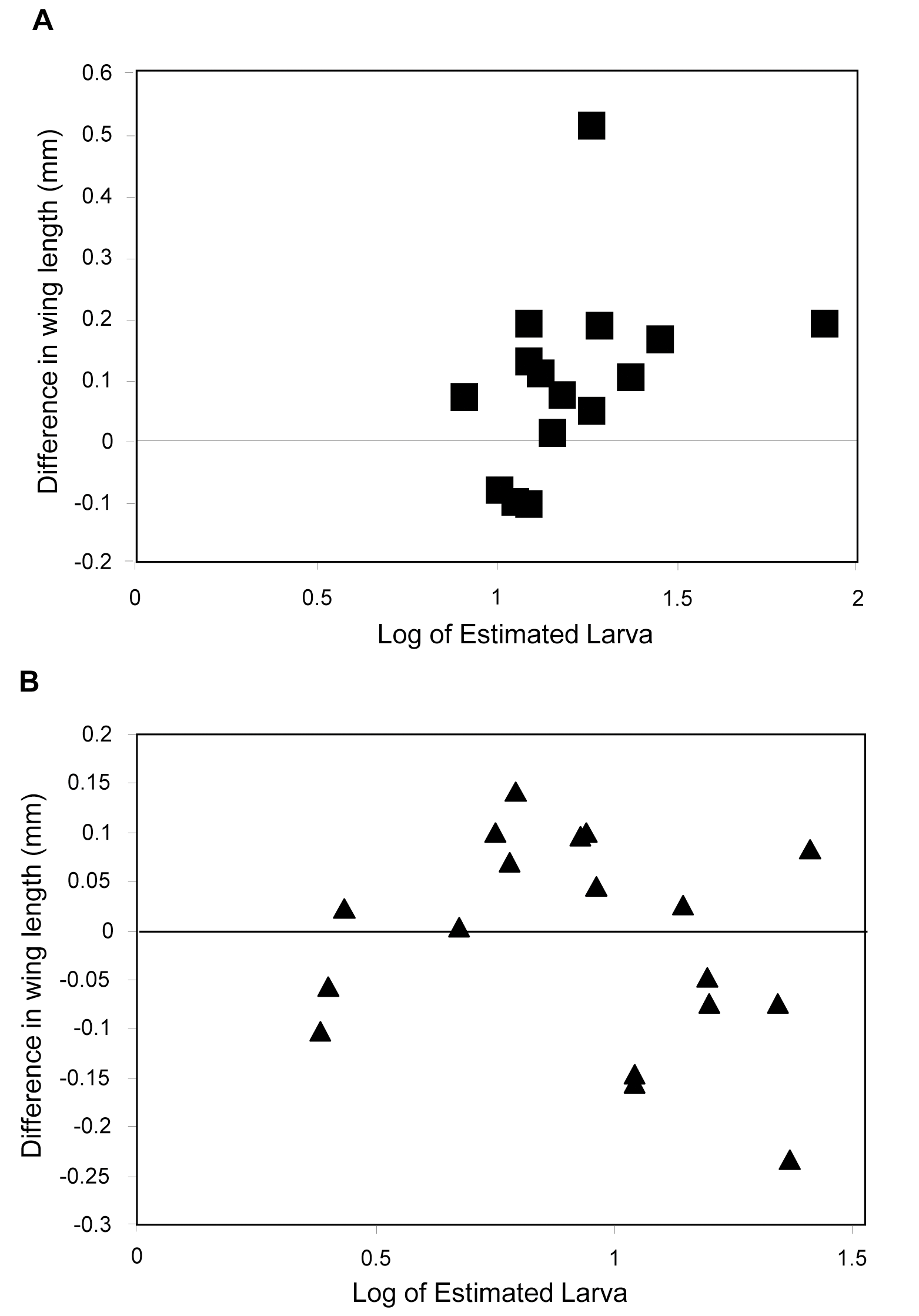

Supplement: Figure S2 — Effect of estimated larval density on male wing length (mm). Relationship between the difference in male wing length between the LA treatment and LP treatment for each container and the log of estimated larval density for both 1× density containers (A) and 10× density containers (B). (TIF) [file pone.0035959.s002.tif]

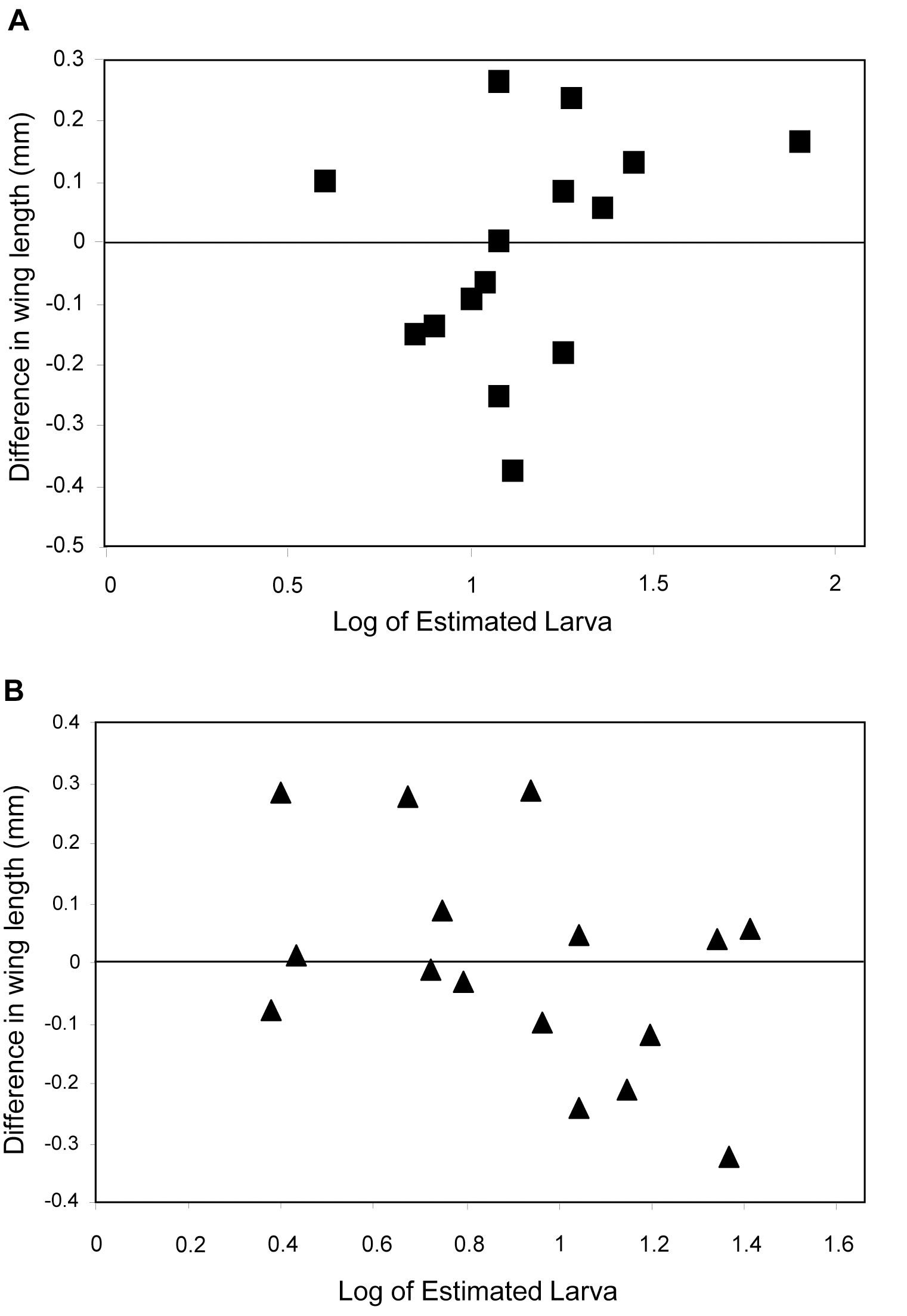

Supplement: Figure S3 — Effect of estimated larval density on female wing length (mm). Relationship between the difference in female wing length between the LA treatment and LP treatment for each container and the log of estimated larval density for both 1× density containers (A) and 10× density containers (B). (TIF) [file pone.0035959.s003.tif]
